# Yin-and-Yang of mTORC1/C2 in Angelman syndrome mice

**DOI:** 10.18632/oncotarget.4298

**Published:** 2015-05-27

**Authors:** Xiaoning Bi, Jiandong Sun, Michel Baudry

**Affiliations:** Western University of Health Sciences, Pomona, CA, USA

The mammalian/mechanistic target of rapamycin (mTOR) exists in two complexes, mTORC1 and mTORC2. Both mTORC1 and mTORC2 contain the mTOR kinase and three other common components: mLST8, deptor, and Tti1/Tel. Additionally, mTORC1 contains PRAS40 and raptor, while mTORC2 contains mSIN1, protor, and rictor [1]. Through integration of a variety of signals, including trophic factors, energy statuses, and NMDA and metabotropic glutamate receptors, mTOR plays critical roles in both brain development and synaptic plasticity. Although a wealth of knowledge has been accumulated regarding the function and regulation of mTORC1, much less information is available for mTORC2 due to its insensitivity to rapamycin. Recent studies, performed mostly with cell lines, have suggested that mTORC1 is activated by growth factors, amino acids, oxygen, and energy statuses, while mTORC2 is mainly stimulated by growth factors.

**Figure 1:**
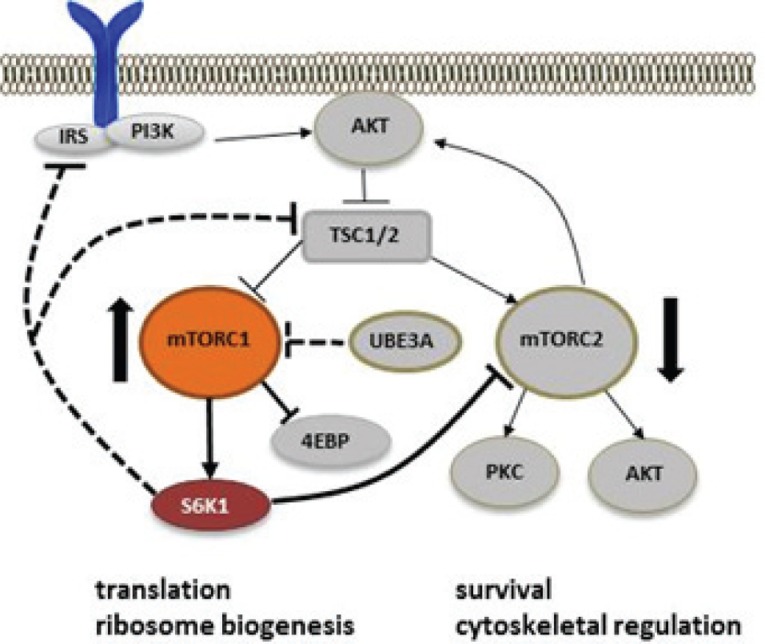
Abnormal mTOR signaling in the cerebellum of Angelman syndrome mice Maternal Ube3A deficiency results in overactivation of mTORC1, which induces mTORC2 inhibition possibly through S6K1-mediated inhibition of insulin receptor substrate (IRS), TSC1/2 complex, and mTORC2 regulator rictor. Dashed lines indicate potential regulations; thin lines indicate reduced activity in AS mice.

Our recent studies have revealed that mTOR signaling is abnormal in the cerebellum of the mouse model for Angelman syndrome (AS) [2]. AS is a genetic neurodevelopmental disorder characterized by severe developmental delay, language and cognition deficits, motor impairment and a happy demeanor. Several lines of evidence have established deficiency in expression of the maternally inherited *UBE3A* gene as the cause for AS [3]. The AS mice have maternal Ube3A deficiency and display the major phenotypes of AS, including memory and motor deficits, and impairment in synaptic plasticity [4, 5]. In our recent study, we found that while mTORC1 activity is increased in the cerebellum of AS mice, mTORC2 activation is reduced. Moreover, increased mTORC1 activation was associated with inhibition of TSC2, which, together with TSC1 and TBC1D7, forms the major inhibitory regulator of mTORC1. This result was surprising since experiments with cell lines have shown TSC2 undergoes UBE3A-dependent degradation. However, we found that TSC2 inhibition was due to increased phosphorylation of an inhibitory site mediated by a rapamycin-sensitive kinase. The imbalance between mTORC1 and mTORC2 is reminiscent to what has been reported in cells lacking the TSC1/2 complex, where inappropriate overactivation of mTORC1 blocks mTORC2-mediated AKT phosphorylation in response to growth factors. Persistent mTORC1 activation is generally postulated to inhibit AKT via its downstream kinase, S6K1, which phosphorylates and inhibits either insulin receptor substrate-1 or members of the mTORC2 complex, rictor at Thr1135 and mSIN1 at Thr86 and Thr398. However, further studies showed that, in some cases, TSC1/2 deficiency results in mTORC2 inhibition in an mTORC1-independent manner, indicating that the TSC1/2 complex may directly stimulate mTORC2 [6]. We found that S6K1-mediated inhibitory phosphorylation of rictor was also increased in AS mice [2]. Thus, reduced mTORC2 activity in AS mice could be a consequence of either decreased stimulation from TSC2 or increased inhibition of rictor by S6K1. Since rapamycin treatment corrected both lower AKT phosphorylation and TSC2 inhibition, mTORC1-S6K1 overactivation is likely a key step underlying abnormal mTOR regulation in AS mice (Fig. 1). How Ube3A deficiency results in abnormal mTOR signaling is not completely clear. It is likely that Ube3A normally imposes a constitutive suppression of mTORC1; lack of Ube3A would then set in motion the abnormal regulation of the mTOR pathway, since rapamycin treatment normalized the activities of both mTORC1 and mTORC2 activities. Rapamycin treatment also corrected abnormalities in dendritic spine morphology of Purkinje neurons and motor function in AS mice [2], indicating that imbalanced mTORC1 and mTORC2 activation contributes, at least in part, to motor dysfunction in AS. Of notice, a recent study has shown that knockout of rictor, either in whole brain or specifically in Purkinje neurons, resulted in changes in neuronal morphology, especially in Purkinje neurons, in a PKC-dependent manner [7]. Another study showed that conditional deletion of rictor in postnatal forebrain excitatory neurons selectively impaired both long-term memory and long-term synaptic plasticity, due to impaired actin polymerization [8]. These results indicate that mTORC2 plays important roles in maintaining neuronal morphology through regulation of the cytoskeletal network. A balanced mTORC1 and mTORC2 activation, with the former controlling protein synthesis and cell proliferation, and the latter regulating cytoskeleton remodeling and cell survival, is necessary for the brain to develop typical circuits and function at optimal levels.

Our results also raise additional questions. For instance, it is well known that mTORC2 activates mTORC1 through AKT phosphorylation, yet our results showed mTORC1 overactivation, despite reduced mTORC2-AKT activation. Could this runaway mTORC1 activation due to an ongoing autophosphorylation? Are TSC2 inhibition or other unidentified factors involved? We showed that mTORC2 inhibition was due to mTORC1S6K1-mediated inhibitory phosphorylation of rictor and possibly TSC2 as well. It remains to be determined how general this regulation is in different brain regions, and whether additional mutual interactions occur at different levels of mTOR signaling cascades. Finally, it is noteworthy that mTOR activity is increased in Fragile X, tuberous sclerosis, and Down's syndromes, but decreased in Rett's syndrome (although these syndromes all show various degrees of intellectual disability). However, in all these variants of autism syndrome disorders, it is not clear whether alterations in mTOR signaling are due to modifications of mTORC1, mTORC2, or both. Further studies directed at these issues should provide a better understanding of these developmental neurologic disorders and reveal potential new therapeutic targets.
